# Impaired Endothelial Nitric Oxide Synthase Homodimer Formation Triggers Development of Transplant Vasculopathy - Insights from a Murine Aortic Transplantation Model

**DOI:** 10.1038/srep37917

**Published:** 2016-11-24

**Authors:** Rupert Oberhuber, Gregor Riede, Benno Cardini, David Bernhard, Barbara Messner, Katrin Watschinger, Christina Steger, Gerald Brandacher, Johann Pratschke, Georg Golderer, Ernst R. Werner, Manuel Maglione

**Affiliations:** 1 Centre of Operative Medicine, Department of Visceral, Transplant and Thoracic Surgery, Medical University Innsbruck, Innsbruck, Austria; 2 Cardiac Surgery Research Laboratory, University Clinic for Cardiac Surgery, Medical University Innsbruck, Innsbruck, Austria; 3 Cardiac Surgery Research Laboratory, Department of Surgery, Vienna Medical University, Austria; 4 Division of Biological Chemistry, Biocenter, Medical University Innsbruck, Innsbruck, Austria; 5 Institute of Pathology, Academic Teaching Hospital Feldkirch, Feldkirch, Austria; 6 Department of Plastic and Reconstructive Surgery, Vascularized Composite Allotransplantation (VCA) Laboratory, Johns Hopkins University School of Medicine, Baltimore, Maryland, USA; 7 Department of General-, Visceral- and Transplantation Surgery, Charité, Campus Virchow Klinikum, Berlin, Germany

## Abstract

Transplant vasculopathy (TV) represents a major obstacle to long-term graft survival and correlates with severity of ischemia reperfusion injury (IRI). Donor administration of the nitric oxide synthases (NOS) co-factor tetrahydrobiopterin has been shown to prevent IRI. Herein, we analysed whether tetrahydrobiopterin is also involved in TV development. Using a fully allogeneic mismatched (BALB/c to C57BL/6) murine aortic transplantation model grafts subjected to long cold ischemia time developed severe TV with intimal hyperplasia (α-smooth muscle actin positive cells in the neointima) and endothelial activation (increased P-selectin expression). Donor pretreatment with tetrahydrobiopterin significantly minimised these changes resulting in only marginal TV development. Severe TV observed in the non-treated group was associated with increased protein oxidation and increased occurrence of endothelial NOS monomers in the aortic grafts already during graft procurement. Tetrahydrobiopterin supplementation of the donor prevented all these early oxidative changes in the graft. Non-treated allogeneic grafts without cold ischemia time and syngeneic grafts did not develop any TV. We identified early protein oxidation and impaired endothelial NOS homodimer formation as plausible mechanistic explanation for the crucial role of IRI in triggering TV in transplanted aortic grafts. Therefore, targeting endothelial NOS in the donor represents a promising strategy to minimise TV.

Solid organ transplantation has become the treatment of choice for patients with end stage organ failure. However, excellent short-term graft survival does currently not mirror long-term outcome, as transplant vasculopathy (TV) continues to be its primary obstacle^1^,^2^,^3^. TV is a multifactorial process. Both, alloimmune and non-alloimmune factors influence occurrence and severity of TV, which finally leads to vascular intimal hyperplastic lesions, the so-called neointima. Exact pathomechanisms are still unknown, effective prevention is not available^4^. Important non-alloimmune factors are donor age, hypertension, hyperlipidemia and pre-existing diabetes, and drug-related side effects (e.g. cytomegalovirus infection, nephrotoxicity and new-onset diabetes after transplantation)^3^,^5^, however, there is some evidence that TV severity is predetermined by the degree of ischemia reperfusion injury (IRI) of the graft occurring in the process of transplantation^3^,^6^,^7^. IRI results in loss of endothelial cell integrity with upregulated expression of proinflammatory cytokines and adhesion molecules^8^,^9^. As a consequence, immune cells start invading the graft perpetuating a constant inflammatory process resulting ultimately in transplant vasculopathy featuring neointimal smooth muscle cell expansion, formation of extracellular matrix and intimal fibrosis^10^.

Ischemia and, paradoxically, the resumption of blood flow during graft reperfusion cause a dramatic increase of superoxide (O_2_^−^·) and peroxynitrite (ONOO^−^) in endothelial cells^11^,^12^. This initial oxidative burst has been shown to deplete endogenous antioxidants like tetrahydrobiopterin (BH4)^13^,^14^,^15^. BH4 is also an essential co-factor of the nitric oxide synthases (NOS) with profound impact on the structure of all three NOS isoforms, stabilising their active homodimeric form and increasing their substrate affinity^16^,^17^. With the depletion of intracellular BH4 by oxidative stress NOS start producing reactive oxygen species rather than nitric oxide, the so-called NOS uncoupling^15^,^17^,^18^,^19^. This finally leads to ONOO^−^ formation and ends into a vicious cycle of excessive oxygen radical formation, antioxidant consumption and tissue destruction^17^,^20^,^21^,^22^.

Recently, we observed in a syngeneic setting, using murine pancreas transplantation model, that BH4 pretreatment of the donor immediately before organ procurement prevented lethal IRI^18^,^23^. Since IRI has been observed to be an essential trigger of TV development, we wanted to investigate whether and how the same BH4 pretreatment protocol of the donor would exert beneficial effects on development of TV. Even though ideal for IRI analyses, the previously used pancreas transplantation model is not suited for long-term observations due to progressive fibrosis of the parenchyma^24^. Therefore, in this study we used a fully allogeneic mismatched aortic transplantation model. This mouse model, with the aortic interpositioning graft transplanted into the common carotid artery of the recipient animal, is a well established mouse model with TV occurring 4 weeks following transplantation^25^,^26^.

## Results

### Neointima formation is minimised by BH4 supplementation

**Figure 1:**
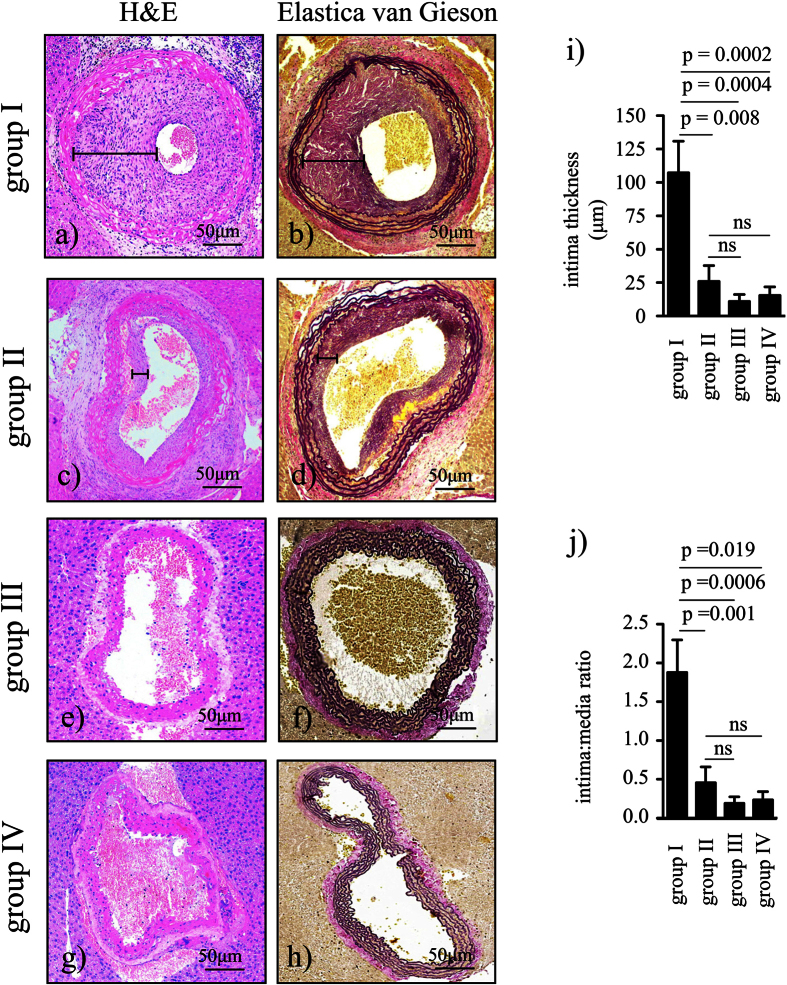
Neointima formation. Aortic grafts were taken from tetrahydrobiopterin (BH4) - treated or non-treated donors, subjected or not subjected to 24 h CIT, and reperfused for 4 weeks. H&E and Elastica van Gieson staining: (a+b) allogeneic non-treated with CIT (group I, n = 10), (c+d) allogeneic BH4 – treated with CIT (group II, n = 9), (e+f) allogeneic non-treated without CIT (group III, n = 9), (g+h) syngeneic control group with CIT (group IV, n = 5). Closed bars in (**a**–**d**) indicate the neointima. Black-staining in (**b**,**d**,**f**,**h**) indicates elastic fibers of the vessel wall. Bar graphs showing (**i**) intima thickness (μm) and (**j**) the intima:media ratio. Results are expressed as mean ± SEM. ns = not significant.

Four weeks following transplantation we observed prominent neointima formation in non-treated allogeneic grafts subjected to 24 h cold ischemia time (CIT) (group I, n = 10) Fig. 1a,b). In contrast, BH4 pretreatment (group II, n = 9) resulted in significantly less intima thickness and intima:media ratio compared to the non-treated allogeneic group (p = 0.008, p = 0.001, respectively; Fig. 1c,d,i,j). Of note, allogeneic grafts without CIT (group III, n = 9) and the syngeneic controls (group IV, n = 5) had also significantly less intima thickness and intima:media ratio compared to the non-treated allogeneic group (group I vs group III: p = 0.0004, p = 0.0006, respectively; group I vs group IV: p = 0.0002, p = 0.019, respectively; Fig. 1e–j). Findings were, however, comparable with the BH4 treated group (p = ns; Fig. 1a,b,e–j).

### Expression of P-selectin is prevented by BH4 supplementation

**Figure 2:**
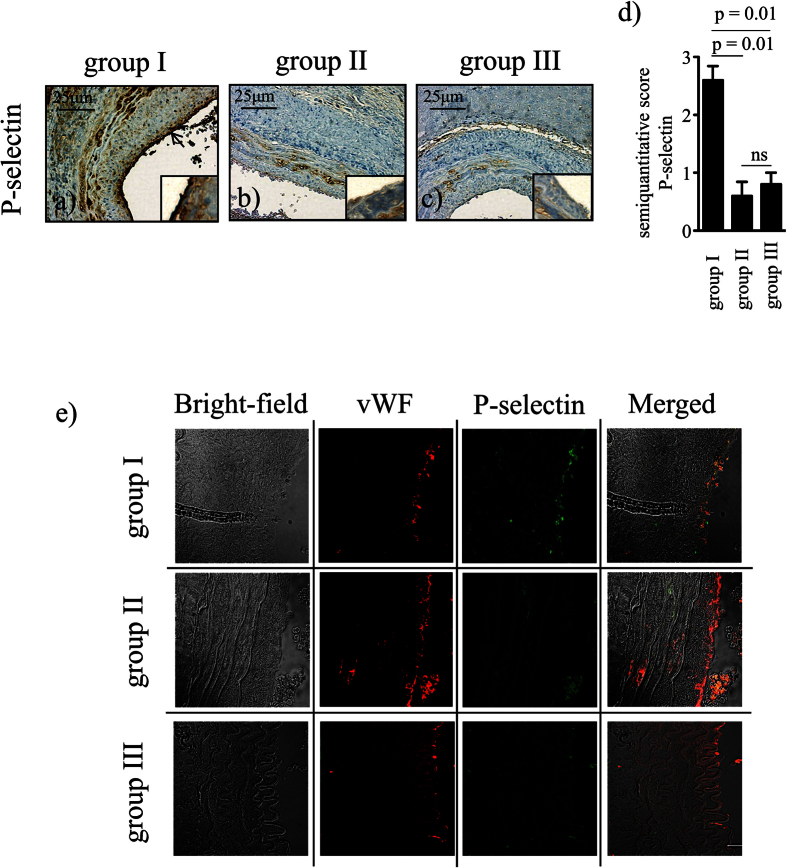
Adhesion-molecule expression. Aortic grafts were taken from tetrahydrobiopterin (BH4) - treated or non-treated donors, subjected or not subjected to 24 h CIT, and reperfused for 4 weeks: (**a**) allogeneic non-treated with CIT (group I, n = 5), (**b**) allogeneic BH4 – treated with CIT (group II, n = 5), (**c**) allogeneic non-treated without CIT (group III, n = 5). Sections were stained for P-selectin (brown; black arrow in (**a**) indicating its luminal expression) and for haematoxylin (nuclei, blue). (**d**) Bar graph showing the semiquantitative score of P-selectin expression. (**e**) Representative photographs of double immunofluorescence against vWF (red) and P-selectin (green) showing the endothelial origin of P-selectin expression in grafts subjected to prolonged CIT. Bright field pictures show the vessel wall structure allowing morphological correlation with the staining. Images of antibody validation for double immunofluorescence staining can be found as Supplementary Fig. S2. Results are expressed as mean ± SEM. ns = not significant.

Endothelial cell activation is characterised by expression of endothelial P-selectin, a hallmark in TV development^27^,^28^. Semiquantitive analyses of immunohistochemical staining showed significantly increased expression of P-selectin 4 weeks after transplantation in non-treated allogeneic grafts with prolonged CIT (group I, n = 5) compared to allogeneic grafts without CIT (group III, n = 5) as well as compared to BH4 treated grafts (group II, n = 5; p = 0.01 and p = 0.01), respectively; Fig. 2a–d). Using immunofluorescence double staining with the endothelial cell marker von Willebrand Factor (vWF), P-selectin expression was co-localised in these cells (Fig. 2e). Again, significant differences of P-selectin expression between the experimental groups could be seen with the immunofluorescence double staining (see Supplementary Fig. S1).

### Expression of α-smooth muscle actin is prevented by BH4 pretreatment

**Figure 3:**
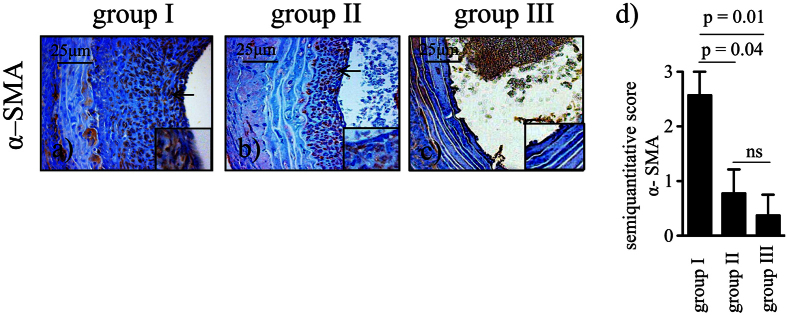
α-SMA positive neointimal formation. Aortic grafts were taken from tetrahydrobiopterin (BH4) - treated or non-treated donors, subjected or not subjected to 24 h CIT, and reperfused for 4 weeks: (**a**) allogeneic non-treated with CIT (group I, n = 10), (**b**) allogeneic BH4 – treated with CIT (group II, n = 9), (**c**) allogeneic non-treated without CIT (group III, n = 9). Sections were stained for α-SMA (brown; black arrows in (**a**,**b**) indicating its expression in the neointima) and for haematoxylin (nuclei, blue). (**d**) Bar graph showing the semiquantitative score of α-SMA expression. Results are expressed as mean ± SEM. ns = not significant.

Another hallmark of TV is the accumulation of α-smooth muscle actin (α-SMA) positive cells within hyperplastic intimal lesions^29^. Similarly to endothelial P-selectin expression, we observed high expression of α–SMA in non-treated allogeneic grafts with CIT (group I, n = 10). Again, not only allogeneic grafts without CIT (group III, n = 9), but also BH4 treated allogeneic grafts (group II, n = 9) showed significantly less α-SMA positive cells (p = 0.01 and p = 0.04, respectively; Fig. 3).

### Infiltration of CD4, CD8 and CD68 positive cells is not influenced by BH4 supplementation

**Figure 4:**
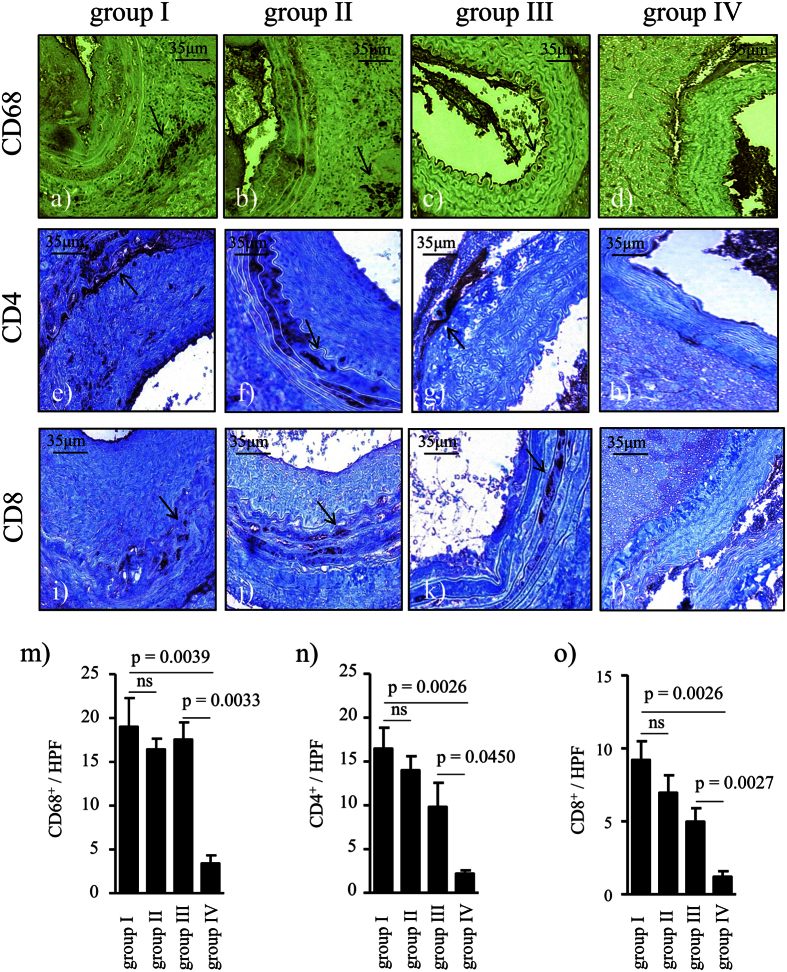
Infiltration with inflammatory cells. Aortic grafts were taken from tetrahydrobiopterin (BH4) - treated or non-treated donors, subjected to 24 h CIT, and reperfused for 4 weeks: (**a**,**e**,**i**) Allogeneic non-treated with CIT (group I, n = 10), (**b**,**f**,**j**) allogeneic BH4 – treated with CIT (group II, n = 9), (**c**,**g**,**k**) allogeneic non-treated without CIT (group III, n = 9), (**d**,**h**,**l**) syngeneic control group with CIT (group IV, n = 5). Sections were stained for CD68 (**a**–**d**), CD4 (**e**–**h**) and CD8 (**i**–**l**) (brown; black arrows in the allogeneic groups indicating stained inflammatory cells) and for methylene green (**a**–**d**; nuclei, green) and for haematoxylin (**e**–**l**; nuclei, blue). Representative images of antibody validation for immunohistochemical staining can be found as Supplementary Fig. S3. (**m**,**n**,**o**) Bar graphs showing the quantitative analysis of inflammatory cell infiltration. Results are expressed as mean ± SEM. ns = not significant.

CD4 and CD8 positive inflammatory cells as well as CD68 positive macrophages are crucial players in TV development^30^,^31^. All allogeneic groups (group I, n = 10, group II, n = 9, and group III, n = 9) showed inflammatory cell infiltration of CD4, CD8 and CD68 positive cells 4 weeks after transplantation, whereas only in the syngeneic controls (group IV, n = 5) no infiltration was detected (Fig. 4).

### Protein oxidation and endothelial Nitric Oxide Synthase (eNOS) monomerisation is prevented by BH4 supplementation

**Figure 5:**
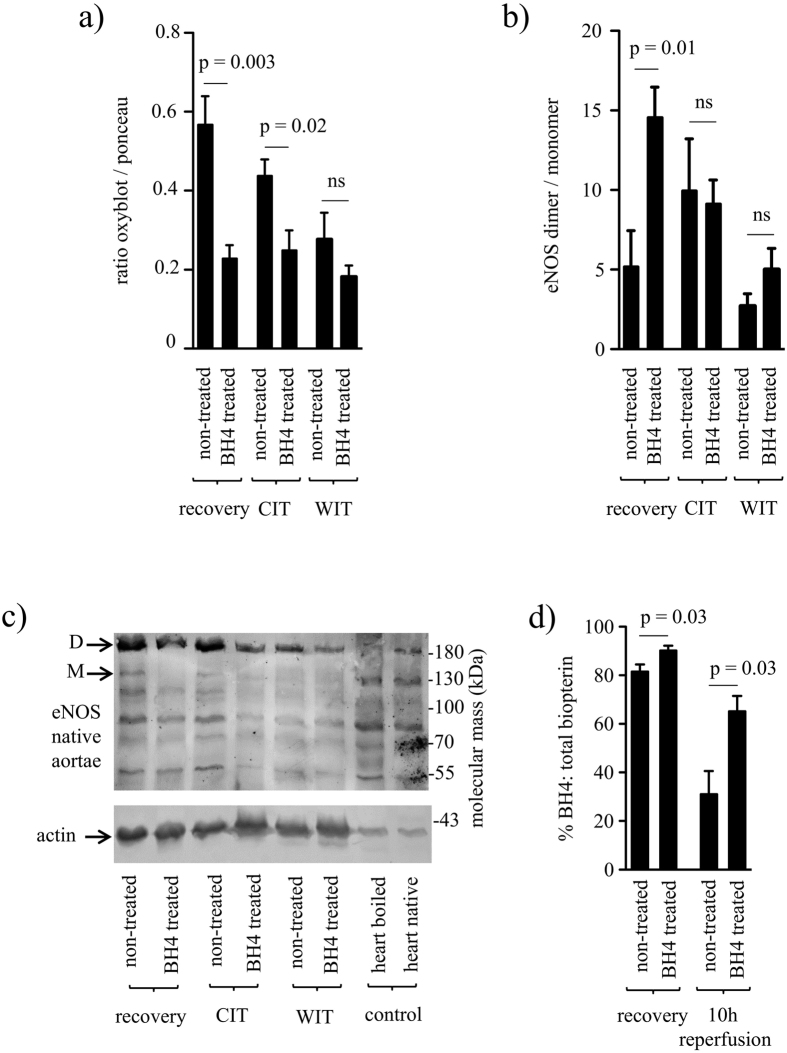
Protein oxidation, eNOS monomer formation and biopterin levels. Aortic grafts were taken from tetrahydrobiopterin (BH4) - treated or non-treated donors, at different time points (at the time of graft procurement ≙ “recovery”, following 24 h CIT ≙ “CIT”, following 24 h CIT plus 45 min WIT ≙ “WIT”, and following 24 h CIT plus 45 min WIT and 10 h of graft reperfusion ≙ “10 h rep”) and assessed for protein oxidation, eNOS dimerisation as well as biopterin content. (**a**) Bar graph showing the ratio between densitometric values of the Oxyblot^®^ bands and those stained with Ponceau red in order to quantify the level of protein oxidation at distinct time points (n = 5/group for each time point). Representative images of protein oxidation and Ponceau red staining blots can be found as Supplementary Fig. S4. (**b**) Quantification of eNOS dimers and monomers in aortas at distinct time points (n = 5/group for each time point). (**c**) Representative blot for determination of eNOS dimers and monomers by western blot at distinct time points in native (non-boiled) aortas. Boiled and native (non-boiled) mouse heart tissue samples served as controls. D = dimer, M = monomer. (**d**) Bar graph showing BH4-to-total biopterin ratios in aortas at distinct time points (n = 5/group for each time point). Results are expressed as mean ± SEM. ns = not significant.

BH4 is known to be essential for stabilising the active, NO producing, dimeric form of eNOS^16^. In contrast, in its monomeric form eNOS generates radical oxygen species promoting oxidative stress^32^. We therefore assessed protein oxidation status and the amount of eNOS monomers. Oxyblot^®^ analysis revealed that already at the time of graft recovery, aortic grafts from non-treated animals showed significantly higher protein oxidation compared to grafts from BH4-treated donor animals (n = 5/group; p = 0.003; Fig. 5a). After 24 h CIT differences in protein oxidation were still significant (n =  = 5/group; p = 0.02; Fig. 5a), whereas after additional 45 min of warm ischemia time (WIT) observed differences in protein oxidation did not reach statistical significance anymore (n = 5/group; p = ns; Fig. 5a).

In parallel to the significantly increased protein oxidation, a significantly lower eNOS dimer to monomer ratio was measured at the time of graft recovery in grafts from non-treated donors compared to the BH4 treated group (n = 5/group; p = 0.01; Fig. 5b). Following 24 h CIT as well as following 24 h and 45 min WIT no significant differences were observed (n = 5/group for each time point; p = ns). This is best visible by the amount of monomer which is much lower in BH4 treated aortas. A representative Western Blot is shown in Fig. 5c.

### Biopterin concentrations within aortic tissue are increased following donor BH4 supplementation

The relationship between active tetrahydro-compound BH4 and non-active dihydro-compound, i.e. the BH4-to-BH2 (dihydrobiopterin) ratio, rather than absolute concentrations of BH4, has been shown to be accountable for normal eNOS function^33^. Measurements of biopterin concentrations revealed significantly higher concentrations of the active tetrahydro-compound in the aorta of treated animals compared to non-treated animals, as mirrored by significantly higher BH4-to-total biopterin ratios at the time of graft recovery as well as 10 h following graft reperfusion (n = 5/group for each time point; p = 0.03 for both time points; Fig. 5d).

## Discussion

This work confirms our hypothesis that a single BH4 supplementation to the donor immediately before organ procurement prevents TV in a fully mismatched, murine allogeneic aorta transplantation model. Additional major findings of this study are (1) the observation that in non-treated allogeneic aortic grafts prolonged CIT is crucial for occurrence of severe TV, and (2) the identification of two non-alloimmune key events happening already during organ procurement, and which seem to trigger severe TV development: BH4 depletion and increased occurrence of inactive eNOS monomers in the graft.

Our results fit very well with the clinical observation that the degree of IRI drastically influences TV development, and are in line with the injury hypothesis where nonantigen-dependent pathways play a pivotal role in graft deterioration^2^,^3^,^6^,^34^. These data confirm our hypothesis that there exists a crucial initial event triggering alloatherogenesis. Living kidney transplantation represents an ideal example for the importance of IRI. Despite similar alloimmune prerequisites graft survival is significantly better in living kidney compared to cadaveric kidney recipients^35^,^36^. One critical difference between these two transplantation strategies is the extremely short cold ischemia time. Also the degree of IRI-associated early microcirculatory disruption in kidney grafts has been shown to be predictive for occurrence of TV and interstitial fibrosis/tubular atrophy (IF/TA)^37^. Similarly, in liver transplantation IRI is a major source of long-term morbidity and mortality, significantly influencing the development of microangiopathic ischemia type biliary lesions occurring months or even years after transplantation^38^. Recently, a linear correlation between duration of CIT and the extent of TV has also been shown in a rat cardiac transplant model^39^. Taken together, severity of IRI crucially determines further graft development. However, there is ongoing controversy whether prolonged CIT per se induces neointima formation (also in a syngeneic setting) or whether the allogeneic response is mandatory for neointima formation, and which are the triggering events initiating TV development^40^,^41^,^42^.

In a murine pancreas transplantation model we showed in a syngeneic setting that long CIT leads to lethal graft injury in the recipients. This could only be prevented by BH4 pretreatment of the donor. However, due to the high amount of active proteases in the pancreas a further dissection of the underlying mechanism was not possible^18^. In the current study, we analysed in an aortic transplantation model the influence of CIT on TV development. The novelty consists on the one hand in the better suited model for analysing the hypothesised protective effect of BH4 in the early stages of the organ transplantation process, and on the other hand, in the allogeneic setting, allowing to analyse the impact of BH4 treatment in the presence of the alloimmune response.

Aortic interpositioning models represent together with heterotopic heart transplantation models accepted mouse models for studying TV^43^. Despite the advantage of transplanting a vascularised organ, heart transplantation models have several drawbacks compared to aortic transplantation models: (i) the heterogeneity of TV in the transplanted heart that results in the need for larger numbers of grafts per group^44^, (ii) the necessity of immunosuppressive treatment of the recipient animals that may modify TV dynamics and (iii) also the likelihood of chronic ischemia in models using the carotid artery due to the inadequacy of the arterial pressure to enable normal coronary perfusion^45^. In contrast, a major drawback described in the aortic interpositioning model is the replacement of donor endothelial and smooth muscle cells by host cells after several weeks following severe rejection^46^. Acting on the assumption that in this study key events happen already during organ procurement we opted for the aortic interpositioning model because of the above mentioned advantages. Results achieved in this study have to be interpreted on the basis of the used aortic interpositioning model.

Four weeks post transplantation we observed dramatically lower P-selectin expression and lower α-SMA expression within neointimal lesions in the allogeneic, treated group compared to the allogeneic, non-treated group. P-selectin and α–SMA are two hallmarks of TV. Vascular wall remodelling in TV is characterized by accumulation of α–SMA positive cells within the intima instead of the medial muscle layer, where, as a consequence of medial degradation, α–SMA positive cells are replaced by scar tissue^10^. In addition, P-selectin is known to promote smooth muscle cell (SMC) migration into the vascular wall, thereby, supporting neointima formation^47^.

Interestingly, in contrast to these two endothelial cell activation and TV markers, BH4 pretreatment of the donor did not influence the infiltration of CD4 and CD8 positive inflammatory cells as well as of CD68 positive macrophages, all cells described to be involved in TV^30^,^31^. This points to the importance of early nonantigen-dependent events on top of antigen-dependent reactions for the development of TV. In fact, neither CIT alone, as shown in the syngeneic control group, nor the mere alloimmune cell infiltration observed in the allogeneic grafts without CIT triggered severe TV. Hence, IRI seems to be as mandatory as the alloimmune reaction for TV occurrence initiating probably a vascular damage as triggering event, which is then sustained by the allogeneic response on top. However, we cannot exclude development of TV in an allogeneic setting without CIT later on after the selected endpoint of 4 weeks used in this study. In this regard, it has been shown that late after transplantation alloimmune factors gain more importance for TV development^3^,^48^.

The observation that BH4 treatment of the donor prevents TV development 4 weeks following transplantation supports the hypothesis that the trigger event takes place on a very early stage of the transplant procedure^6^,^34^.

BH4 is a powerful naturally occurring antioxidative agent and an essential co-factor of the NOS. BH4 availability is crucial for formation and stabilisation of active NOS homodimers. Decreased BH4 levels have been associated with increased occurrence of inactive eNOS monomers, consistent with NOS uncoupling^32^. This refers to the generation of superoxide rather than nitric oxide (NO) by the NOS enzyme due to the electron transfer being uncoupled during the enzymatic reaction. NOS uncoupling results in endothelial dysfunction^49^, a pathomechanism which is linked to numerous cardiovascular diseases and is also involved in IRI following solid organ transplantation^17^,^50^,^51^,^52^. Previous studies showed that prevention of IRI by BH4 is specific for this compound and related to its NOS co-factor activity rather than to its antioxidant capacity^23^,^52^.

Analyses of intragraft protein oxidation and BH4 levels clearly show a high degree of deleterious oxidative damage and BH4 depletion at the time of graft recovery. Both, however, are significantly attenuated by BH4 treatment of donor. Interestingly, this happens even though BH4 is applied only 2 minutes before organ procurement. The depletion of BH4 is likely to result from direct oxidative degradation as a consequence of the oxidative stress during graft procurement. In fact, under an altered redox state, BH4 availability is impaired as BH4 biosynthesis is crucially dependent from a normal cellular redox state^16^.

In parallel, at the time of graft recovery, retrieved aortic grafts of non-treated donors had a higher amount of inactive eNOS monomers compared to grafts from treated animals, in which active eNOS dimers were predominant. This suggests that intragraft depletion of BH4 which is demonstrated by a reduced BH4-to-BH2 ratio within aortic grafts results in increased occurrence of inactive eNOS monomers, a mechanism known to be at the basis of endothelial dysfunction^17^,^32^,^53^. In fact, a reduced BH4-to-BH2 ratio, rather than absolute concentrations of BH4, is accountable for normal eNOS function^33^. So, single administration of BH4 to the donor prevented decreased BH4-to-BH2 and as a consequence eNOS dysfunction, which in our opinion is of crucial importance for TV occurrence. These results are also in line with the disappointing results of oral BH4 treatment in patients suffering from coronary artery disease. Despite elevated BH4 levels BH4-to BH2 ratio did not increase. Therefore, no effects were seen on endothelial function, vascular superoxide production and eNOS coupling^54^.

The proposed pathomechanism based on BH4 depletion and increased occurrence of inactive eNOS monomers is also in line with the knowledge that NO inhibits the expression of adhesion molecules^55^, and with the observation that in a balloon angioplasty model with prominent endothelial injury increased generation of NO prevented intimal thickening and restenosis of the vessel by inhibiting SMC proliferation^56^,^57^. BH4 and NO homeostasis have also been shown to play a crucial role in Cyclosporine A (CyA) induced vasomotor dysfunction proposing this pathway as a potential therapeutic strategy to prevent CyA induced vascular injury in transplant recipients^58^. However, the crucial difference between our study and the previously published models is the alloimmune response. Here, prevention of the non-alloimmune, early oxidative endothelial injury in the graft prevented also the development of alloimmune, chronic rejection. So, destabilisation of the active eNOS homodimer, consistent with endothelial dysfunction, appears to be crucial for triggering TV development.

Pretreatment of the donor before organ procurement is not new to the organ transplantation field. Recently, therapeutic hypothermia of the donor has been proposed as a promising strategy to protect recipients from delayed graft function following kidney transplantation^59^. Hence, from a clinical point of view, pretreatment of the donor with BH4 would be definitely feasible, and application of BH4 minutes before starting organ procurement would even ease logistical requirements. In our previous work comparing different BH4 administration strategies like supplementation of the perfusion/storage solution, recipient or donor treatment, only donor treatment showed significant protection from IRI^18^. This is in line with the current results showing crucial events occurring already during organ procurement (treatment of the recipient “misses the target”) and also with observation that the equilibrative nucleoside transporter ENT1, which is responsible for BH4 uptake, is temperature-dependent. The lower the temperature the lower the intracellular BH4 uptake (currently, organs are perfused and stored under hypothermic conditions)^60^,^61^. Donor pretreatment seems, therefore, to be currently the only option to prevent IRI and, as a consequence, TV by BH4. Whether the emergence of normothermic perfusion strategies^62^ offers new treatment opportunities for BH4 has to be further analysed.

In conclusion, we observed a direct correlation between increased eNOS monomer amounts due to BH4 depletion at the time of graft procurement and severe TV development later on. This represents a plausible mechanistic explanation of the clinical observation, that severity of IRI crucially influences TV occurrence and graft deterioration. These results open new therapeutic scenarios in the prevention of TV, currently a major challenge in solid organ transplantation. Pretreatment of the donor with exogenous BH4 or other agents targeting eNOS in the graft might represent in this regard a promising future strategy.

## Methods

### Murine Aortic Transplantation

Ten- to twelve week old male BALB/c (H2d) and C57Bl/6 (H2b) mice (Charles River Sulzfeld, Germany) served as size–matched donor-recipient pairs in a fully MHC mismatched model, except for one group where C57Bl/6 (H2b) mice served as, both, donors and recipients. Thoracic aortic transplantation was performed using a cuff technique as previously described^25^ under clean, but not sterile conditions, using an operating microscope with 7–70x magnification (SZ-STU2, Olympus Inc. Japan). Animals were anesthetised with an intramuscular (i.m.) injection of xylazine (5 mg/kg b.w.) and ketamine (100 mg/kg b.w.). In order to prevent postoperative pain, buprenorphin (0.1 mg/kg b.w.) was administered every 12 hours s.c. for the first 5 days and carprofen (Rimadyl^®^) 4 mg/kg b.w. every 12 hours s.c. for the first week. Break-off criteria included a weight loss of more than 10–15% compared to weight at surgery date, apathy, crippling or a very bent back. If animals met one of these criteria they were sacrificed using terminal isoflurane inhalation before reaching the clinical end point. The perfusion solution Custodiol (HTK, Dr. Franz Köhler Chemie GmbH, Alsbach–Hähnlein, Germany) was used. Animals were housed in a barrier pathogen free facility and received human care in compliance with the “Principals of Laboratory Animal Care” formulated by the National Society for Medical Research and the “Guide for the Care and Use of Laboratory Animals” prepared by the National Academy of Sciences and published by the National Institutes of Health (NIH Publication no. 86–23, revised 1985). All methods and specimen acquisition performed as well as the care of the animals used were in accordance with accepted guidelines for animal research. Experiments were approved by the Austrian Ministry of Education, Science, and Culture (BMWF-66011_0063-II_10b_2010).

### Experimental Design

Aortic grafts were subjected to 24 h of preservation on ice CIT, if not otherwise specified, and to 45 min “rewarming” in the implantation site before reperfusion (WIT). Occurrence of TV was evaluated by histopathology, immunohistochemistry and immunofluorescence 4 weeks following transplantation, a sufficient time span for neointima development^25^,^26^. Study groups consisted of non-treated allogeneic donors (group I; n = 10), BH4 treated allogeneic donors (group II; n = 9), non-treated allogeneic donors without CIT (group III; n = 9), non-treated syngeneic donors (group IV; n = 5). Treatment consisted of 50 mg/kg b.w. BH4 ((6 R)-5,6,7,8-tetrahydro-L-biopterin dihydrochloride; Schircks Laboratories, Jona, Switzerland), dissolved in sterile infusion-grade phosphate buffered saline (PBS) and administered to the donor i.m. 2 min before graft recovery^18^.

In a separate approach, BH4 tissue levels from treated and non-treated donors were measured in aortic grafts at the time of graft procurement, following 24 h CIT plus 45 min WIT, and following 10 h of graft reperfusion (n = 5 per group). Similarly, Oxyblot^®^ analysis and measurement of eNOS dimerisation were conducted in aortic grafts immediately after graft recovery, following 24 h CIT, and in aortic grafts subjected to 24 h of CIT plus 45 min WIT (n = 5 per group).

### Histopathology

Grafts were embedded in liver tissue from the recipient mouse to improve tissue processing. Specimens were fixed in 4% buffered formaldehyde and embedded in paraffin. Separated sections were stained with haematoxylin & eosin (H&E) or Elastica van Gieson. All vessel sections were digitally photographed at 10-fold magnification using a digital camera (Canon Power Shot G5, Canon, Berlin Germany). Captured images were divided into 4 quadrants, and in each quadrant 4 measurements of intima and media thickness were performed. 3 cross sections per animal were assessed, resulting in 48 measurements per animal. Analyses were carried out using Image J 1.32j-software for Java (National Institutes of Health, USA).

### Immunohistochemistry

The following primary antibodies were used on paraffin-embedded sections: anti α-SMA (Clone 1A4, Code M0851, dilution 1:100, Dakocytomation, Glostrup, Denmark), anti-CD62P (P-selectin, NCL-CD62P-367, dilution 1:50, Novocastra Laboratories Ltd, Newcastle upon Tyne, United Kingdom), anti-CD68 (Clone PG-M1, code M0876, dilution 1:100, Dakocytomation, Glostrup Denmark), anti-CD8 (Clone CD8/144B, code M7103, dilution 1:50, Dakocytomation, Glostrup, Denmark) and anti-CD4 (NCL-L-CD4–1F6, dilution 1:100, Novocastra Laboratories Ltd, Newcastle upon Tyne, United Kingdom). To avoid unspecific binding slides were pre-incubated with the Rodent Block of the Mouse on Mouse Polymer Kit (Abcam, Cambridge, United Kingdom). The Vectastain Elite ABC Kit Universal (Vector Laboratories, Burlingame, Ca, USA) was applied according to the instructions. The activity of endogenous peroxidase was blocked with 3% hydrogen peroxide (H_2_O_2_) in methanol. The sections were visualised with the Vector DAB Substrate Kit for Peroxidase (SK-4100; Vector Laboratories Inc., Burlingame, California, USA) and counterstained with haematoxylin. P-selectin as well as α-SMA were assessed based on a previously established four-graded classification^63^,^64^. A blinded scientist performed semiquantitative analysis of cell infiltration in 5 non-overlapping high power fields (HPF).

### Double Immunofluorescence Staining

Following fixation in 4% paraformaldehyde, dehydration, and embedding in paraffin, cross sections were prepared. After deparaffinisation with xylol, heat-mediated antigen retrieval in Tris/Ethylenediaminetetraacetic Acid (EDTA) buffer (pH = 9) was performed. Unspecific binding sites were blocked using 1% Bovine Serum Albumin (BSA) in Tris-Buffered Saline and Tween (TBS-T). Afterwards, the slides were incubated with the primary anti-P-selectin antibody (M-20, Santa Cruz Biotechnology, Heidelberg, Germany, dilution 1:50) and the anti- vWF, Dakocytomation, Glostrup, Denmark, dilution 1:400). Next, slides were washed with TBS-T and incubated with the secondary antibodies: Alexa-488 for the P-selectin-antibody (1:1000, Life Technologies, Vienna, Austria) and Alexa-546 for the vWF-antibody (1:1000, Life Technologies, Vienna, Austria). After three washing steps, tissues were mounted in Pro Long Antifade Gold (Life Technologies, Vienna, Austria) and image acquisition was conducted using a LSM 510 Meta attached to an Axioplan 2 imaging MOT using ZEN software 2008 (Zeiss, Germany). Aortic sections of ApoE -/- fed at high fat diet (for 12 weeks) were used as positive controls, as the development of atherosclerotic lesions in this well established mouse model is accompanied by the endothelium - specific up-regulation of P-selectin^64^.

### Oxyblot^®^ - Analysis

Oxidative modification of proteins was analysed using the Oxyblot^®^ technology (Merck Millipore, Vienna, Austria), which is based on the detection of carbonyl groups introduced into proteins by oxidation reactions. Briefly, after mechanical breakup of vascular tissue, preparation of proteins in lysis buffer and ultrasound treatment, the non-soluble fraction in the extract was removed. The supernatant was then used for derivatisation of carbonyl groups in the protein side chains to 2,4-dinitrophenylhydrazone (DNP-hydrazone) by reaction with 2,4-dinitrophenylhydrazine (DNPH). The DNP-derivatised protein samples were then separated by polyacrylamide gel electrophoresis (PAGE) followed by blotting onto a nitrocellulose membrane. Thereafter, membranes were incubated with primary antibody, specific to the DNP moiety of the proteins (rabbit anti-DNP antibody), and then followed by incubation with a horseradish peroxidase-antibody conjugate (secondary antibody: goat anti-rabbit horseradish peroxidase (HRP) antibody). The membranes were then treated with Enhanced chemiluminescence (ECL, Thermo Fisher Scientific, Vienna, Austria), followed by the detection of the signal by exposure to x-ray films. Following development and fixation of films, bands were imaged, and quantified using a standard imaging system and normalised to the total protein amount of the respective lane stained with a 0.1% (w/v) Ponceau S solution with 5% Acetic acid (Sigma Aldrich, Vienna, Austria).

### Analysis of eNOS monomer dimer ratio by low temperature PAGE and Western blot

Aortae were homogenised with an Ultra Turrax (IKA, Staufen, Germany) in 100 μl 50 mM TrisHCl, pH 7.4, containing 150 mM NaCl, 0.5% Triton X-100 and a protease inhibitor mix (GE Healthcare, Vienna, Austria), centrifuged at 16000 × g and 4 °C, and protein determined by the Bradford assay (Biorad, Vienna, Austria). Homogenates were mixed with standard Laemmli sample buffer at 4 °C with the standard heating step omitted to preserve the dimer. To calibrate the blot for eNOS monomer size a boiled and a non-boiled mouse heart tissue sample was run in parallel^65^. 80 μg of protein were separated by standard Laemmli conditions with precooled 7.5% gels and buffers in an ice bath in the cold room. Proteins were then blotted for 1 h at room temperature to a polyvinylidene fluoride (PVDF) membrane, blocked with milk powder in PBS-T (0.1%) for 2 h, and stained for eNOS with sc-654 rabbit polyclonal IgG (Santa Cruz Biotechnology, Heidelberg, Germany) and for actin with MAP 1501 R (Merck-Millipore, Vienna, Austria). After washing with PBS-T (0.1%), staining with fluorescent secondary antibodies Cy5 anti-rabbit and Cy3 anti-mouse (both from GE Healthcare) membranes were scanned with a three colour fluorescence scanner (Typhoon 9410, GE Healthcare) and the intensity of the bands quantified by the Imagequant software (GE Healthcare).

### Biopterin Measurements by High Performance Liquid Chromatography (HPLC)

Intragraft BH4 concentrations were measured by a method modified from Fukushima and Nixon^66^. BH4 concentrations were calculated as difference of biopterin concentration under acidic and basic oxidation conditions. Dihydrobiopterin plus biopterin (BH2 + B) concentrations were calculated as difference between total intragraft biopterin and intragraft BH4 concentration.

### Statistical Analysis

Results are expressed as mean ± standard error of the mean (SEM). Statistical analysis was performed using GraphPad Prism 5 (GraphPad Software, La Jolla, CA, USA). When comparing multiple groups Kruskal-Wallis test was applied. If statistical significance was achieved, all pairs were compared among each other using the Mann-Whitney-U and the Bonferroni Multiple Comparison post hoc test. A p value of <0.05 was considered to be of statistical significance.

## Additional Information

**How to cite this article**: Oberhuber, R. *et al*. Impaired Endothelial Nitric Oxide Synthase Homodimer Formation Triggers Development of Transplant Vasculopathy - Insights from a Murine Aortic Transplantation Model. *Sci. Rep*. **6**, 37917; doi: 10.1038/srep37917 (2016).

**Publisher's note:** Springer Nature remains neutral with regard to jurisdictional claims in published maps and institutional affiliations.

## Supplementary Material

Supplementary Information
