# Supplementary material for: Impaired Endothelial Nitric Oxide Synthase Homodimer Formation Triggers Development of Transplant Vasculopathy - Insights from a Murine Aortic Transplantation Model
Source: Sci Rep. 2016 Nov 24;6:37917. doi: 10.1038/srep37917 (PMC5121662; doi:10.1038/srep37917)
Supplement: Supplementary Information [file srep37917-s1.doc]

***Supplementary Information***

**Impaired Endothelial Nitric Oxide Synthase Homodimer Formation Triggers Development of Transplant Vasculopathy -** **Insights from a Murine Aortic Transplantation Model**

Oberhuber Rupert MD^1^, Riede Gregor MD^1^, Cardini Benno MD^1^, Bernhard David PhD^2^, Messner Barbara PhD^3^, Watschinger Katrin PhD^4^, Steger Christina MD^5^, Brandacher Gerald MD^1,6^, Pratschke Johann MD^1,7^, Golderer Georg PhD^4^, Werner Ernst R DSc^4*^ and Maglione Manuel MD^1*^

^1^Centre of Operative Medicine, Department of Visceral, Transplant and Thoracic Surgery, Medical University Innsbruck, Innsbruck, Austria; ^2^Cardiac Surgery Research Laboratory, University Clinic for Cardiac Surgery, Medical University Innsbruck, Innsbruck, Austria ^3^Cardiac Surgery Research Laboratory, Department of Surgery, Vienna Medical University, Austria; ^4^Division of Biological Chemistry, Biocenter, Medical University Innsbruck, Innsbruck, Austria; ^5^Institute of Pathology, Academic Teaching Hospital Feldkirch, Feldkirch, Austria; ^6^Department of Plastic and Reconstructive Surgery, Vascularized Composite Allotransplantation (VCA) Laboratory, Johns Hopkins University School of Medicine, Baltimore, Maryland, USA, ^7^Department of General-, Visceral- and Transplantation Surgery, Charité, Campus Virchow Klinikum, Berlin, Germany; ^*^corresponding authors

**Figures:**

**Supplementary Figure S1**


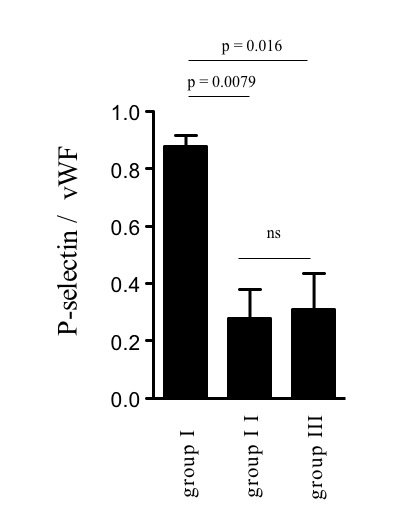


**Legends:**

**Supplementary Figure S1: P-selectin expression based on immunofluorescence double staining.**

Aortic grafts were taken from tetrahydrobiopterin (BH4) - treated or non-treated donors, subjected or not subjected to 24 h CIT, and reperfused for 4 weeks.

This bar graph shows the evaluation of the immunofluorescence double staining with P-selectin and the endothelial cell marker von Willebrand Factor (vWF) in the different study groups (group I, n = 5, allogeneic non-treated with CIT; group II, n = 5, allogeneic BH4 – treated with CIT; group III, n = 5, allogeneic non-treated without CIT). Image analysis for quantification of P-selectin/vWF expression was performed by a blinded scientist using the Photoshop CC Version 2015.5 software (Adobe Systems, Mountain View, CA, USA) as previously described.^1^

Results are expressed as mean ± SEM. ns = not significant.

**Supplementary Figure S2**


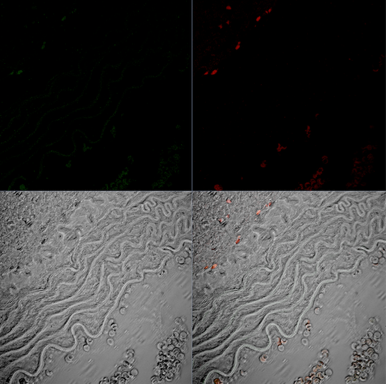


P-selectin

vWF

a)


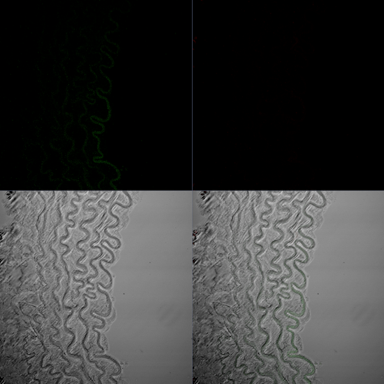


P-selectin

vWF

b)


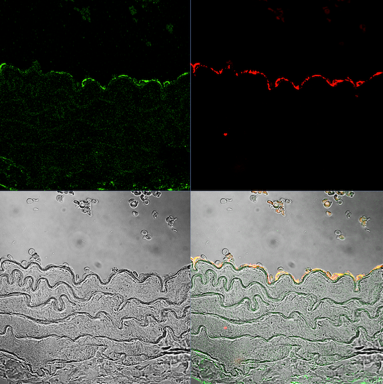


P-selectin

vWF

c)

**Supplementary Figure S2: Antibody validation for double immunofluorescence staining against vWF (red) and P-selectin (green).**

a) Isotype controls for P-selectin (mouse IgG1) and vWF (rabbit polyclonal IgG) on murine artic transplantation grafts

b) Secondary antibody controls for P-selectin (Alexa 488 donkey anti goat) and vWF (Alexa 546 goat anti rabbit) on murine artic transplantation grafts

c) Aorta of an ApoE- knockout mouse 12 weeks on high fat diet (acting as positive control for endothelial activation)

Bright field pictures in the lower part of the images show the vessel wall structure allowing morphological correlation with the staining.

vWF = von Willebrand Factor

**Supplementary Figure S3**


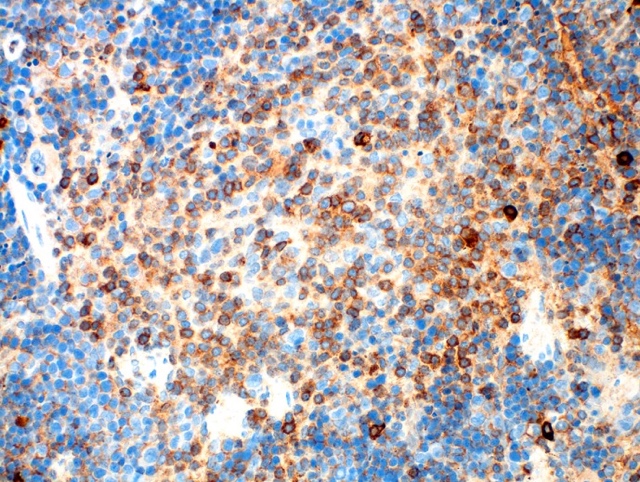


**CD4**


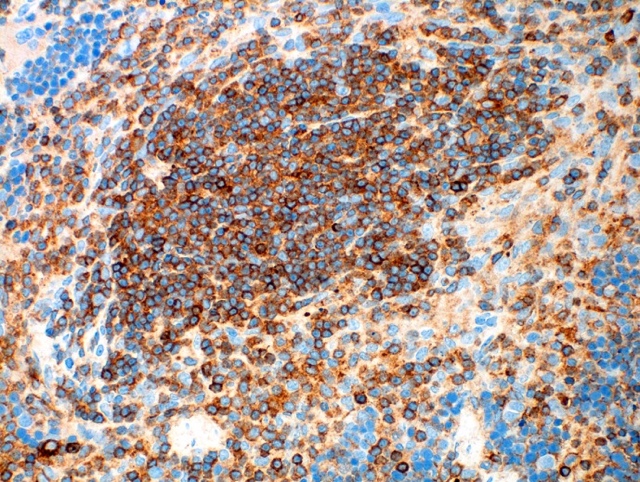


**CD8**


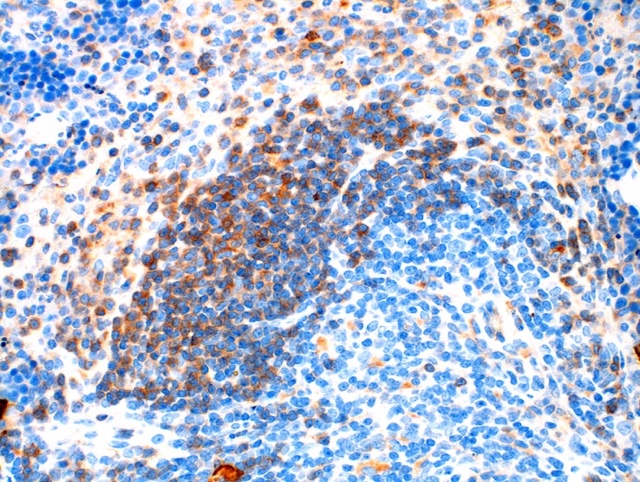


**CD68**


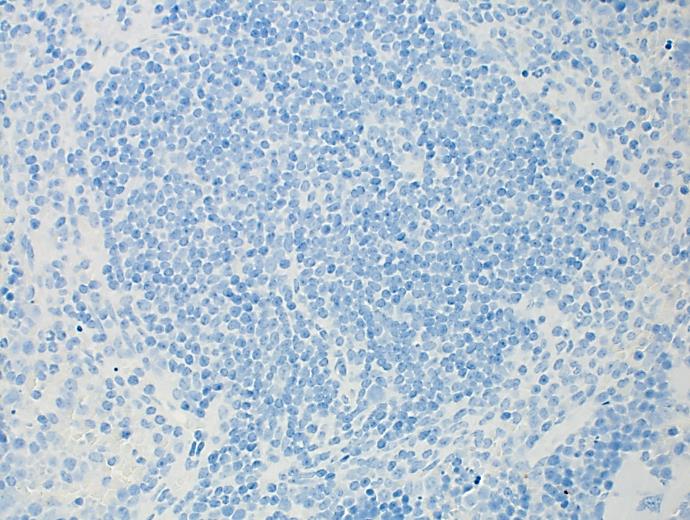


**neg control**

**Supplementary Figure S3: Antibody validation for immunohistochemical staining of CD4, CD8 and CD68.**

Spleens were taken from non treated donor mice (BALB/c). These images show sections of paraffin embedded spleen specimens stained for CD4, CD8, CD68 (brown) and for hematoxylin (nuclei, blue). All sections show a white pulp area of murine spleen. As negative control only the secondary antibody and a counter staining with hematoxylin was used.

**Supplementary Figure S4**


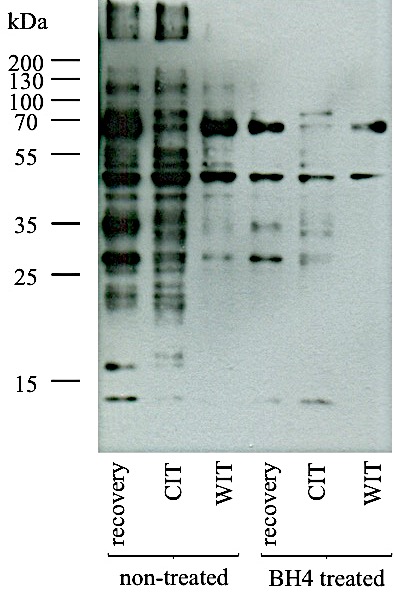


**a)**


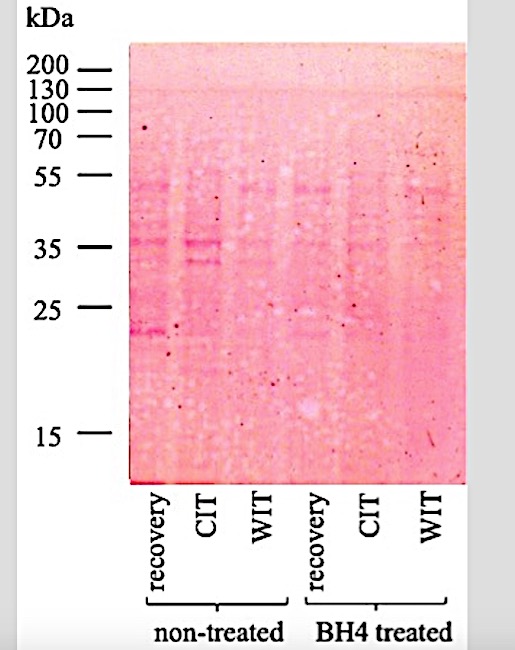


**b)**

**Supplementary Figure S4:** **Protein oxidation and Ponceau S staining blots.** Aortic grafts were taken from tetrahydrobiopterin (BH4) - treated or non-treated donors, at different time points (at the time of graft procurement ≙ “recovery”, following 24 h CIT ≙ “CIT”, following 24 h CIT plus 45 min WIT ≙ “WIT”) and assessed for protein oxidation.

Images show representative blots for (a) determination of protein oxidation products by Oxyblot® at distinct time points, as well as (b) the corresponding Ponceau red staining. n = 5/group for each time point.

**References**

1 Blunder, *S. et a*l. Characteristics of TAV- and BAV-associated thoracic aortic aneurysms--smooth muscle cell biology, expression profiling, and histological analyses*. Atheroscleros*i**s 2**20, 355-361, doi:10.1016/j.atherosclerosis.2011.11.035 (2012).
